# Preparation and Characterization of Porous Core-Shell Fibers for Slow Release of Tea Polyphenols

**DOI:** 10.3390/polym10020144

**Published:** 2018-02-02

**Authors:** Yaru Wang, Lan Xu

**Affiliations:** National Engineering Laboratory for Modern Silk, College of Textile and Engineering, Soochow University, 199 Ren-ai Road, Suzhou 215123, China; yrwang@stu.suda.edu.cn

**Keywords:** coaxial electrospinning, porous, core-shell, tea polyphenol, drug release

## Abstract

This study focused on the fabrication, characterization, and release properties of electrospun tea polyphenol (TPP) loaded porous core-shell structured fibers. The morphology, structure and properties of the electrospun TPP loaded porous core-shell fibers were investigated by a combination of Fourier transformation infrared spectroscopy (FTIR), scanning electron microscopy (SEM), contact angle (CA) measurements, transmission electron microscopy (TEM), etc. In addition, the cumulative drug release rate of TPP loaded porous core-shell fibers was determined by ultraviolet (UV) spectrophotometer, and the release mechanism was investigated using Fickian diffusion equation, which would provide the theoretical basis for future study. The results showed TPP loaded porous core-shell structured fibers were successfully prepared by coaxial electrospinning, and the porous structure of the core-shell fibers could further enlarge the specific surface area, enhance the hydrophobic properties, and improve the drug release properties.

## 1. Introduction

Drug loaded nanofiber membranes prepared by single electrospinning usually show an initial burst release because of the high concentration of the drug distributed on the nanofiber surface. The core-shell nanofibers, in which the drug is restricted to the core layer, is expected to limit the release of the drug during the initial stage [1]. Coaxial electrospinning is a versatile method for preparing core-shell nanofibers. Drugs can be encapsulated into the core phase by using this technique. With increased efficiency and encapsulation rate, the burst release can be reduced, and drug bioactivity can be protected [2,3]. The current method may find wide applications for controlled release of proteins and tissue [4]. When proper polymer systems are employed, and parameters are adjusted, ideal core-shell structured nanofibers can be produced, and the release behavior can be effectively controlled. Over the past several decades, there has been growing interest in development of coaxial electrospinning in accordance with therapeutic purposes, and the pharmacological or biological properties [5,6].

Due to the nanoporous materials with ultra-high adsorption, high surface energy, high surface activity, and other characteristics [7], nanoporous materials are potentially of great technological interest for the development of chemical engineering, electronic engineering, biological medicines, and other related areas [8,9,10,11,12,13]. Recent research showed that the single electrospinning process can be used to fabricate nanofibers with porous surface by adjusting electrospinning parameters, and the porous structure could promote release of drug [2,14,15].

Tea polyphenol (TPP) has a lot of beneficial effects, such as antioxidant, anticancer, and antibacterial properties [16]. However, TPP is oxidized easily due to light, heat, and oxidants. Therefore, chemical instability of TPP is the major constraint to its use as therapeutic agent. Therefore, it is important to improve the chemical stability and enhance the efficacy of TPP for its use as therapeutic agent [17]. In recent years, incorporation of TPP in lipid composite nanoparticles can overcome and resolve this problem. Nanoparticle delivery systems based on chitosan (CS), a natural biomaterial, has been widely applied in pharmaceutical fields to enhance absorption of bioactive compounds. But the pH, ions, digestive enzymes in the gastrointestinal (GI) tract, and mucus layer, impact the properties of nanoparticle delivery system [18].

In this paper, TPP loaded porous core-shell fibers were prepared successfully by controlling coaxial electrospinning parameters. Poly(lactic acid) (PLA) was selected as shell material, because of its excellent biocompatibility and biodegradability [19]. A model drug TPP was loaded in the polyethylene oxide (PEO) via physical blending. The morphology and structure of the core-shell fibers were investigated by scanning electron microscopy (SEM), contact angle (CA) measurements, and transmission electron microscopy (TEM). Successful entrapments of TPP in the core-shell fibers were validated by Fourier transform infrared (FTIR) spectroscopy. The cumulative drug release rate of TPP from the core-shell fibers was measured in Phosphate buffer saline (PBS) for a period of 141 h using ultraviolet (UV) spectrophotometer. The release mechanism of TPP in the core-shell fibers was investigated using Fickian diffusion equation. The results showed the porous structure of the electrospun core-shell fibers further enlarged the specific surface area, enhanced the hydrophobic property, and improved the release property of TPP.

## 2. Experimental

### 2.1. Materials

Poly(lactic acid) (PLA) with an average molecular weight of 100,000 g/mol was supplied by Shenzhen Esun Industrial Co. Ltd. (Shenzhen, China). Tea polyphenols (TPPs) were purchased from Suzhou BIO-NOW Biotech Co. Ltd. (Suzhou, China) (purity: ≥98%). Polyethylene oxide (PEO) with an average molecular weight of 600,000 g/mol, dichloromethane (DCM) (Analytical Reagent) and *N*,*N*-dimethylformamide (DMF) (Analytical Reagent) were supplied by Shanghai Chemical Reagent Co. Ltd. (Shanghai, China). *n*-Butanol was purchased from Wuxi Prospect Chemical Reagent Co. Ltd. (Wuxi, China). Phosphate buffer saline (PBS) (Analytical Reagent, pH7.0) was supplied by Shanghai Beyotime Biological Technology Co. Ltd. (Shanghai, China). All chemicals were used without further purification.

### 2.2. Solution Preparation

All concentration measurements were done in weight by weight (*w*/*w*). PLA shell solutions were prepared with 8 wt % by using mixture of solvents DCM and DMF with various weight ratios of 5:5, 7:3, and 9:1, respectively. PEO core solutions were prepared with 7 wt % by using DMF as the solvent, and 0.05 g TPPs were added into 10 g core solution. The obtained solutions of shell layer and core layer were magnetically stirred for 12 h at room temperature until they became homogeneous.

### 2.3. Fabrication of TPP Loaded Core-Shell Fibers

**Figure 1:**
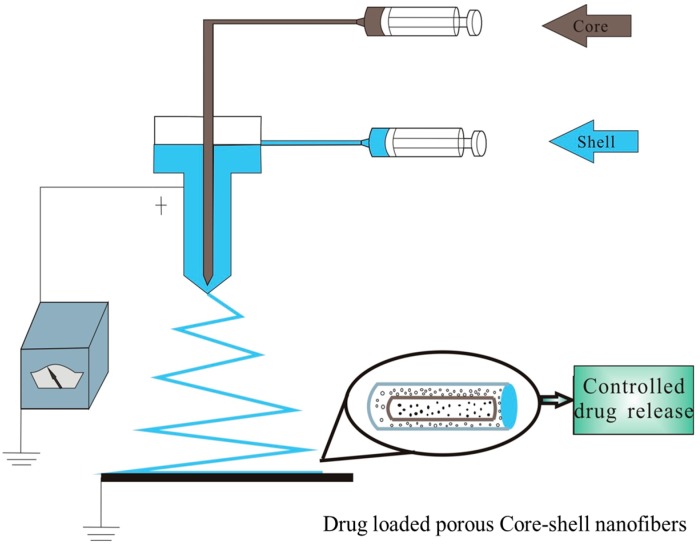
Schematic diagram of the coaxial electrospinning setup.

The coaxial electrospinning setup consisted of a coaxial double spinneret, a grounded collecting plate, two syringe pumps, and a variable DC high-voltage power generator, as shown in Figure 1. The coaxial double spinneret included two concentrically arranged capillaries [20]. A certain amount of the two polymer solutions were contained separately in two plastic syringes connected to the coaxial spinneret, and the flow rate in each capillary was adjusted in a double-way medical syringe pump [21]. The inner and outer diameters of the inner capillary were 0.557 and 0.788 mm, and the inner and outer diameters of the outer capillary were 1.469 and 1.822 mm.

The applied spinning voltage and distance between the tip of the needle and the collector were maintained at 18 kv and 15 cm. The flow rate of shell solution was maintained at a constant rate of 2 mL/h, and the flow rate of core solution was set to 0.05, 0.1, and 0.2 mL/h, respectively. All the coaxial electrospinning processes were carried out at room temperature (25 ± 2 °C) and a relative humidity of (55 ± 5%).

For comparison, TPP/PLA, TPP/PEO, and TPP/PLA/PEO fibers were prepared, respectively, by single electrospinning. TPP/PLA/PEO solutions were prepared by using mixture of PLA shell solutions and PEO core solutions with the weight ratio of 10:1. The applied voltage, flow rate, and collecting distance were maintained at 18 kv, 2 mL/h, and 15 cm. All the electrospinning processes were carried out at room temperature (25 ± 2 °C) and a relative humidity of (55 ± 5%).

## 3. Characterization and Measurement

### 3.1. Scanning Electron Microscopy (SEM)

The morphologies of TPP loaded core-shell fibers were observed by a scanning electron microscopy (SEM, Hitachi S-4800, Tokyo, Japan) at an acceleration voltage of 3 kV. And the diameter distributions of fibers were analyzed using ImageJ software (National Institute of Mental Health, Bethesda, MD, USA).

### 3.2. Transmission Electron Microscopy (TEM)

Transmission electron microscopy (TEM) (FEI Tecnai G-20, FEI Company, Hillsboro, OR, USA) at an acceleration voltage of 200 kV was used to investigate the core-shell structure of the fibers. The samples were prepared by directly depositing a thin layer of fibers onto the TEM-specific copper grids, and were dried in a vacuum oven for 48 h prior to TEM testing.

### 3.3. Fourier Transform Infrared (FTIR) Spectroscopy

The TPP loaded core-shell fibers were characterized through FTIR spectroscopy (Nicolet5700, Thermo Nicolet Company, Waltham, MA, USA). Each spectrum was obtained by the performance of 32 scans, with the wave number ranging from 500 to 4000 cm^−1^.

### 3.4. Porosity Measurement

Electrospun TPP loaded core-shell fiber membranes were immersed into *n*-butanol for 1 h until equilibrium was achieved at room temperature. The porosities of the membranes were measured by weighing them before and after absorption of the *n*-butanol. The porosity (*P*) (%) was calculated by [22]

(1) $$P\%=\frac{\frac{M}{\rho }}{\frac{M}{\rho }+\frac{M_{m}}{\rho_{m}}}\times 100\%$$

where *M* is the mass of *n*-butanol absorbed by the soaked membrane, $M_{m}$ is the mass of the dry membrane, ρ is the density of *n*-butanol, and $\rho_{m}$ is the density of the dry membrane.

### 3.5. Contact Angle (CA) Measurements

The static contact angles (CAs) of TPP loaded core-shell fiber membranes were investigated using a Krüss DSA 100 apparatus (Krüss Company, Hamburg, Germany). A drop of liquid, placed on a flat surface, is photographed, and the image is analyzed to give the angle at the edge. The volume of droplet used for static CA was 6 μL, and the average water CAs were obtained by measuring the same sample at least in five different positions.

### 3.6. In Vitro TTP Release Study

20 mg of TPP loaded core-shell fibers with the different weight ratios (DCM/DMF) were dispersed, respectively, in 3 mL of PBS (pH 7.0), and then placed in 50 mL centrifuge tubes (Shanghai Hongsheng Biotech Co. Ltd., Shanghai, China). The centrifuge tubes containing core-shell fibers were incubated in a shaking incubator (FLY-100/200, Shanghai Shenxian Thermostatic Equipment Factory, Shanghai, China) with a shaking speed of 60 rpm at 37 °C. At specific intervals, 1 mL of the medium was withdrawn for analysis, and 1 mL of fresh PBS was replenished for continuing incubation, in order to keep the PBS liquid fresh. The experiments were performed in triplicate to obtain more accurate results. The cumulative drug release rate of TPP loaded core-shell fibers were determined by UV spectrophotometer (TU-1810, Beijing Purkinje General Instrument Co., Ltd., Beijing, China). And the amount of TPP released was calculated based on a calibration curve of TPP in PBS.

## 4. Results and Discussion

### 4.1. Morphological Characterization of TPP Loaded Core-Shell Fibers (SEM and TEM)

**Figure 2:**
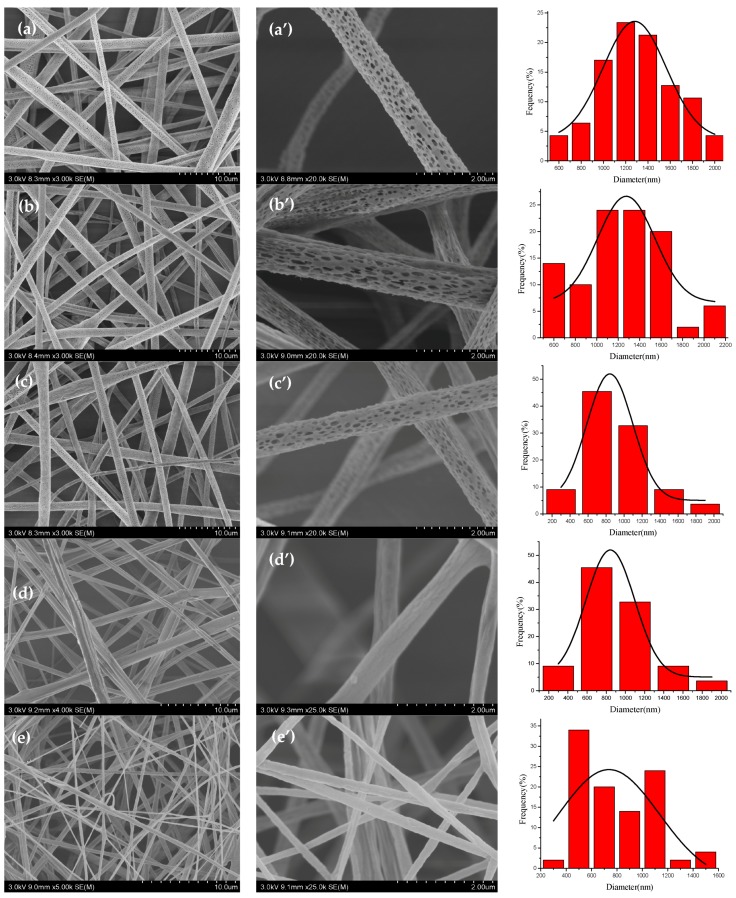
SEM pictures of tea polyphenol (TPP) loaded porous core-shell fibers with the weight ratio 9:1 at core solution flow rates of (**a**,**a’**) 0.05 mL/h; (**b**,**b’**) 0.1 mL/h; and (**c**,**c’**) 0.2 mL/h, respectively, and nonporous core-shell fibers at core solution flow rates of 0.2 mL/h with the weight ratio (DCM/DMF) (**d**,**d’**) 7:3 and (**e**,**e’**) 5:5. The rightmost figures were the according diameter distribution.

The morphologies of TPP loaded core-shell fibers with the different weight ratios (DCM/DMF) in the shell solution were characterized by SEM as shown in Figure 2, and the rightmost figures were the corresponding fiber diameter distribution. The distribution of fiber diameters was determined by measuring 100 fibers selected randomly from the SEM pictures using ImageJ software. Figure 2a–c,a’–c’ and Table 1 showed that as the flow rate of core solution increased, the average diameter of porous TPP loaded core-shell fibers decreased. It could be explained during coaxial electrospinning process that the increase of core flow rate would lead to the increase of the charge density of polymer jets, and lower resistance of the solution, which could be used to further stretch the jet into thinner fibers [8,23]. The ratio of the flow rate of the core and shell is a primary factor affecting the formation of core-shell structured fibers [2]. When the flow rate of the shell solution was 2 mL/h, the solutions have better spinnability [24,25,26]. As summarized in Table 1, the average diameter of core-shell fibers was relatively small at the core solution flow rate of 0.2 mL/h. Therefore, the core solution flow rate of 0.2 mL/h was selected in the following study.

Then Figure 2c–e,c’–e’ showed that with the decrease of the weight ratio (DCM/DMF) in the shell solution, the surface morphology of the core-shell fibers changed from porous (9:1), crack (7:3), to smooth (5:5). And the average diameter of TPP loaded core-shell fibers decreased due to the disappearance of pores [27], as presented in Table 1. There appeared evident pores on the core-shell fiber surface with the weight ratio 9:1. That meant highly volatile solvent utilized in coaxial electrospinning process could create pores on the fibers’ surface [28]. The formation of pores on the jets would waste the amount of energy which, otherwise, could be used to further stretch the jet into even smaller fibers, and result in the increase of the average diameter of porous fibers [8,27].

**Figure 3:**
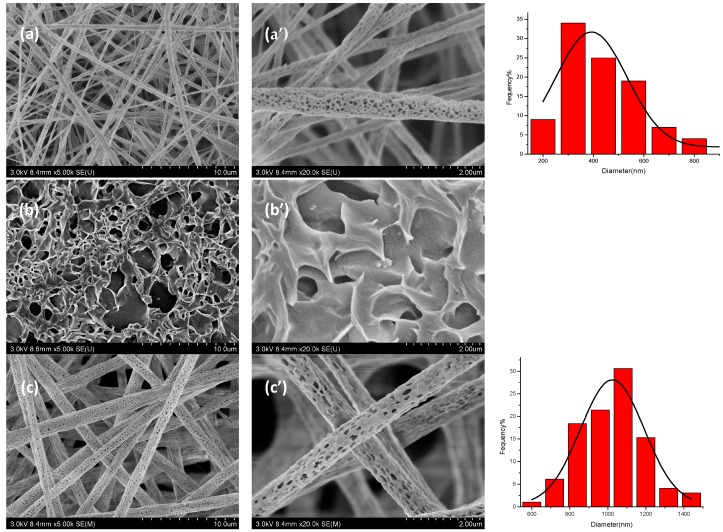
SEM pictures of electrospun porous TPP/ poly(lactic acid) (PLA) (**a**,**a’**), TPP/ polyethylene oxide (PEO) (**b**,**b’**) and TPP/PLA/PEO (**c**,**c’**) fibers. The rightmost figures were the according diameter distribution.

Figure 3 showed that the surface morphologies of electrospun porous TPP/PLA, TPP/PEO, and TPP/PLA/PEO fibers. It could be seen that the PEO solutions have poor spinnability, and TPP/PEO fibers could not be prepared successfully. And as presented in Table 2, with the addition of PEO, the average diameter of TPP loaded fibers increased due to increased average molecular weight [27]. Moreover, the average diameter of non-core-shell fibers was smaller than that of core-shell fibers.

**Figure 4:**
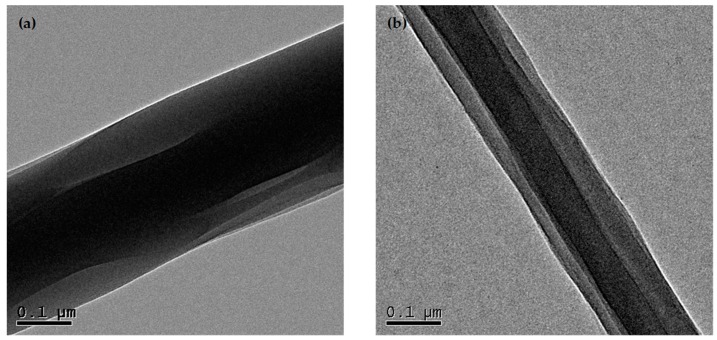
TEM photographs of the TPP loaded core-shell fibers with the different weight ratios (DCM/DMF) ((**a**) 9:1; (**b**) 5:5) at core solution flow rate of 0.2 mL/h.

The core-shell structures of TPP loaded porous and nonporous core-shell fibers were investigated by TEM, as shown in Figure 4.

### 4.2. FTIR Spectrum Analysis

**Figure 5:**
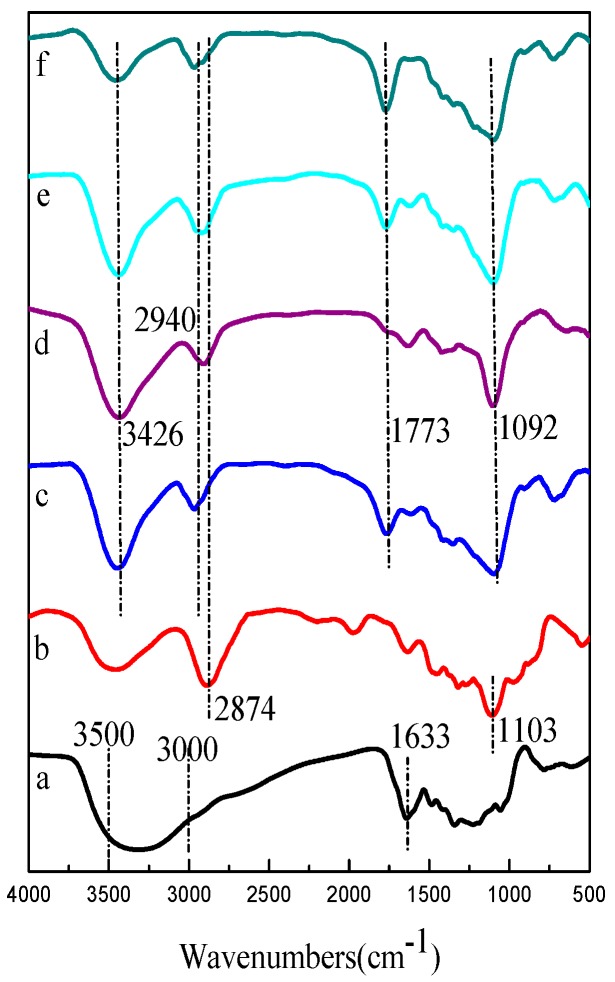
FTIR spectra of TPP (**a**); PEO (**b**); PLA (**c**); and core-shell fibers with the different weight ratios (DCM/DMF) ((**d**) 9:1; (**e**) 7:3; (**f**) 5:5) at core solution flow rate of 0.2 mL/h.

Figure 5 presented the FTIR spectra of TPP, PEO, PLA, and TPP loaded core-shell composite fibers with the different weight ratios (DCM/DMF) at a core solution flow rate of 0.2 mL/h. The FTIR spectrum of TPP (Figure 5 (a)) showed a strong and wide peak corresponding to O–H stretch appeared in the 3000–3500 cm^−1^ region, and a CC stretching peak at 1633 cm^−1^ [29]. The FTIR spectrum of PEO (Figure 5 (b)) exhibited a C–H stretching peak at 2874 cm^−1^ and a COC stretching peak at 1103 cm^−1^ [30]. The FTIR spectra of PLA and core-shell fibers (Figure 5 (c–f)) displayed characteristic absorption bands at 1773 cm^−1^ and 1092 cm^−1^, which represented the backbone ester group of PLA [31]. Figure 5 (c–f) also showed a C–H stretching peak at 2940 cm^−1^ and an O–H stretching peak at 3426 cm^−1^. It could be seen that there is no significant difference in FTIR spectra of PLA and core-shell fibers with different weight ratios (DCM/DMF). Also, the characteristic peaks of TPP and PEO did not appear on the spectra of core-shell fibers, which indicated the absence of TPP and PEO on the surface of core-shell fibers. The FTIR data showed that the core-shell structure of composite fibers, and TPP, was encapsulated in the core-shell fibers.

### 4.3. Porosity Analysis

The Brunauer–Emmett–Teller (BET) surface area of porous fibers has been fully researched in references [32,33,34]. The results reveal that although with a larger diameter the Brunauer–Emmett–Teller (BET) surface area of porous fibers is higher than that of nonporous fibers [33]. And the uniform porous pore size is advantageous and favorable for the drug delivery applications [34]. In this study, the porosities of the PLA fiber membranes and the core-shell fiber membranes were calculated according to Equation (1). The results of porosity measurements were presented in Table 3. It was seen that the porosities of the porous core-shell fiber membranes were relatively high, and that of the porous core-shell fiber membranes at core solution flow rate of 0.1 mL/h was highest. That meant high surface area could result in high porosity. And the variety in membrane porosity was mainly caused by the decreased or increased pore diameter and the uniformity of pore distribution [8].

### 4.4. Contact Angle (CA) Measurements

**Figure 6:**
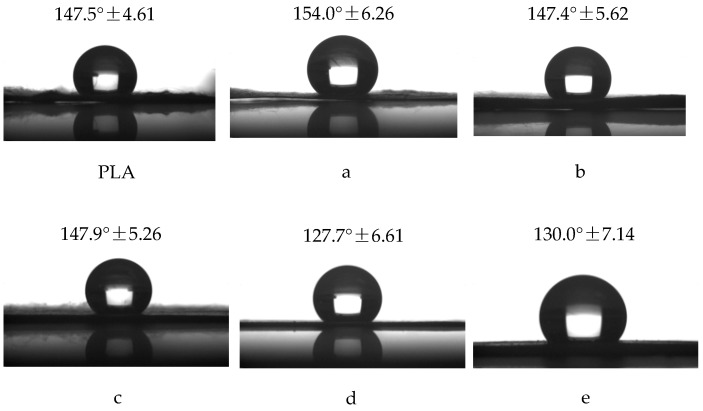
Static contact angle of PLA fibers, porous core-shell fibers with the weight ratio 9:1 at core solution flow rates of (**a**) 0.05 mL/h; (**b**) 0.1 mL/h; and (**c**) 0.2 mL/h, respectively, and nonporous core-shell fibers at core solution flow rates of 0.2 mL/h with the weight ratio (DCM/DMF) (**d**) 7:3 and (**e**) 5:5.

Figure 6 shows the CA values of fiber membranes obtained by single and coaxial electrospinning, respectively. It could be seen that electrospun fiber membranes were hydrophobic materials, due to the hydrophobicity of PLA, and the hydrophobicity of the porous fiber membranes was found to be enhanced. This is because the hierarchical pore structure further enlarges the surface area, and enhances the hydrophobic property of the fiber membranes [35]. In addition, the core layer material is primarily PEO, which is a hydrophilic material. However, the CA values of the core-shell fibers obtained at different flow rates of core solution did not significantly change compared with that of PLA fibers, indicating the absence of PEO on the surface of fibers and the formation of core-shell structured composite fibers.

### 4.5. In Vitro Drug Release Profile

**Figure 7:**
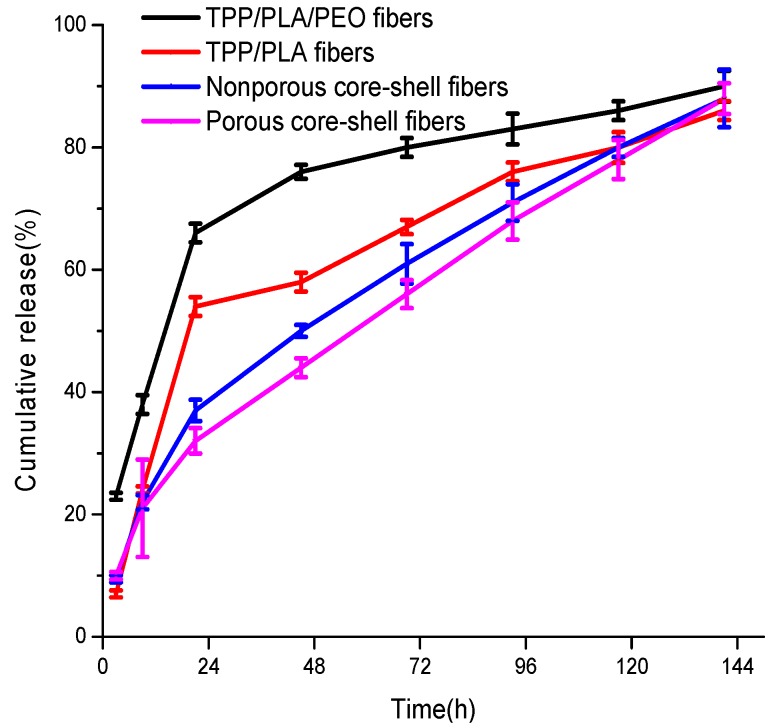
TPP release profiles from porous core-shell, nonporous core-shell, PLA, and PLA/PEO fiber membranes loaded with TPP.

The standard curve of TPP concentration to OD 274 nm was calculated according to *y* = 0.02579*x* + 0.00273 (R = 0.999), where *x* is the concentration of TPP (µg/mL), *y* is the OD 274 nm reading value. The accumulative release dose was calculated based on the standard curve. The release profiles of TPP at 37 °C in PBS (pH 7.0) from porous and nonporous core-shell fiber membranes, which were fabricated at core solution flow rates of 0.2 mL/h, were displayed in Figure 7. The results showed that porous and nonporous core-shell fiber membranes have the same drug release behavior, in which an initial burst release is followed by a constant slow release, up to 88% during 141 h incubation. However, after 21 h, the TPP release of porous core-shell, nonporous core-shell, PLA and PLA/PEO fiber membranes loaded with TPP reached 32%, 37%, 54%, and 66%, respectively. It could be seen that the initial burst release of TPP was prevented by core-shell fibers compared to non-core-shell fibers due to the presence of shell acting as an additional layer [4]. The porous core-shell fibers showed a slower burst release than that of nonporous core-shell fibers, since the porous structure could promote release of drug [36], and PLA/PEO fibers had a faster burst release than that of PLA fibers, due to larger diameters. However, core-shell fibers released lower amounts of TPP than non-core-shell fibers, due to the low solubility of TPP in core solutions [36]. In addition, the substitution of 1 mL of PBS with a fresh PBS solution after each UV measurement would make the measured amount of TPP released lesser than its actual amount [37].

**Figure 8:**
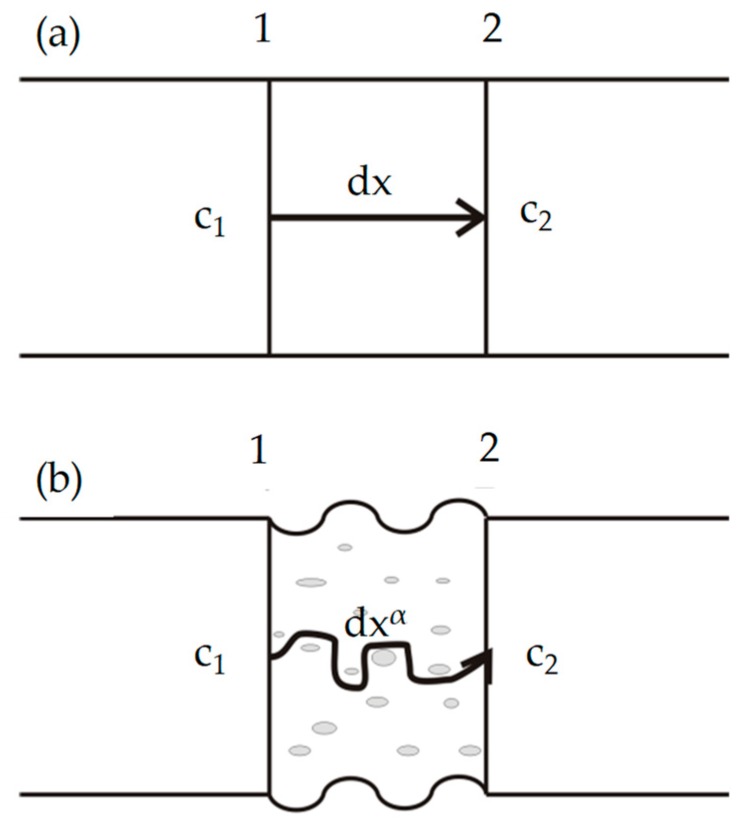
Schematic diagrams of Fickian diffusion of drug from (**a**) nonporous fibers and (**b**) porous fibers.

Drug release kinetics from nonporous fibers could be explained using the following Fickian diffusion equation:

(2) $$J\;=\;-D\frac{dC}{dx}$$

where *J* was the diffusion flux of drug, *D* was the diffusion coefficient of drug, *C* is the volume concentration of drug, *dx* was the diffusion path from Section 1 to Section 2, *dC/dx* was the concentration gradient of drug, and ‘−’ indicated that the diffusion direction was the opposite direction of concentration gradient, that meant, the drug was diffused from high concentration (C1) Section 1 to low concentration (C2) Section 2, as shown in Figure 8a.

Then Figure 8b illustrated the drug release kinetics from porous fibers, and the diffusion path $dx^{\alpha }$ was shown in the arrow. Therefore, Fickian diffusion equation could be written as follows:

(3) $$J\;=\;-D\frac{dC}{dx^{\alpha }}$$

where α was the diffusion path exponent that represented the diffusion mechanism of drug release. Compared with the nonporous fibers, the diffusion path $dx^{\alpha }$ had a longer path than dx, due to the porous structure of the fibers, that was α>1.

According to Equation (3), the concentration gradient of drug would become smaller due to the increase of the diffusion path. Therefore, the diffusion flux would decrease, and result in the slow release of drug. The theoretical results were in good agreement with the experimental data as shown in Figure 6, and showed the porous structure of core-shell fibers could reduce the burst release and better control the drug release.

## 5. Conclusions

TPP loaded porous core-shell fibers, in which the blend of TPP and PEO encapsulated the PLA layer, were fabricated successfully by controlling coaxial electrospinning parameters, such as ratio of solvent and flow rate of core solution. SEM images showed with the increase of high volatility solvent DCM in the mixed shell solvent system, the morphology of the core-shell fibers changed from smooth, crack to porous. And the average diameter of porous core-shell fibers decreased with the flow rate of core solution increased. TEM images displayed the ideal core shell structure of TPP loaded porous and nonporous fibers. FTIR data showed that the absence of PEO on the surface of fibers and the formation of core-shell fibers. CA measurements indicated the hydrophobic property of the core-shell fibers and confirmed the core-shell structure of composite fibers.

The cumulative drug release rates of TPP from the core-shell fibers were measured in PBS for a period of 141 h using UV spectrophotometer. The release mechanism of TPP in the core-shell fibers was investigated using Fickian diffusion equation, and would provide the theoretical basis for future study. The results showed the initial burst release of TPP was prevented by core-shell fibers compared to non-core-shell fibers, and the porous structure of the core-shell fibers could reduce the burst release and better control the drug release.

## Figures and Tables

**Table 1:** Average diameters of TPP loaded core-shell nanofibers.

Sample	Flow Rate of Core Solution (mL/h)	DCM/DMF	Average Diameter ($\bar{D}$) (nm)	Standard Deviation (*σ*) (nm)	Confidence Interval (nm)
Nonporous core-shell fibers	0.2	7:3	782	292.2	±81.1
		5:5	642	238.7	±66.2
Porous core-shell fibers	0.05	9:1	1401	472.0	±130.8
	0.1		1256	397.6	±110.2
	0.2		916	346.7	±91.6

**Table 2:** Average diameters of TPP loaded non-core-shell fibers.

Sample	Flow Rate (mL/h)	DCM/DMF	Average Diameter ($\bar{D}$) (nm)	Standard Deviation (*σ*) (nm)	Confidence Interval (nm)
TPP/PLA fibers	2	9:1	447	166.1	±32.6
TPP/PLA/PEO fibers			1032.18	200.2	±39.2

**Table 3:** Porosities of PLA and core-shell fibers.

Sample	Flow Rate of Core Solution (mL/h)	DCM/DMF	Average Fiber Membrane Quality (g)	Porosity (%)
Non core-shell PLA		9:1	0.0046	76
Porous core-shell fibers	0.01		0.0035	82
	0.1		0.0033	88
	0.2		0.0069	80
Nonporous core-shell fibers	0.2	7:3	0.0138	72
		5:5	0.0096	72

## References

[1] He C.L., Huang Z.M., Han X.J. (2009). Fabrication of drug-loaded electrospun aligned fibrous threads for suture applications. J. Biomed. Mater. Res. Part A.

[2] Nguyen T.T., Ghosh C., Hwang S.G., Chanunpanich N., Park J.S. (2012). Porous core/sheath composite nanofibers fabricated by coaxial electrospinning as a potential mat for drug release system. Int. J. Pharm..

[3] Qin Y., Liu R., Zhao Y., Hu Z., Li X. (2016). Preparation of dipyridamole/polyurethane core–shell nanofibers by coaxial electrospinning for controlled-release antiplatelet application. J. Nanosci. Nanotechnol..

[4] Jiang H., Hu Y., Li Y., Zhao P., Zhu K., Chen W. (2005). A facile technique to prepare biodegradable coaxial electrospun nanofibers for controlled release of bioactive agents. J. Controll. Release.

[5] Ballios B.G., Kooy C.M.D. (2010). A hydrogel-based stem cell delivery system to treat retinal degenerative diseases. Biomaterials.

[6] He M., Xue J., Geng H., Gu H., Chen D., Shi R., Zhang L. (2015). Fibrous guided tissue regeneration membrane loaded with anti-inflammatory agent prepared by coaxial electrospinning for the purpose of controlled release. Appl. Surface Sci..

[7] Xu L., Wu Y., Liu Y. (2010). Electrospun nanoporous materials: Reality, potential and challenges. Mater. Sci. Technol..

[8] Zhao J., Si N., Xu L., Tang X., Song Y., Sun Z. (2016). Experimental and theoretical study on the electrospinning nanoporous fibers process. Mater. Chem. Phys..

[9] Mckee M.G., Long T.E. (2006). Phospholipid nonwoven electrospun membranes. Science.

[10] Dzenis Y.A. (2004). Spinning continues fibers for nanotechnology. Science.

[11] Reneker D.H., Yarin A.L., Hao F., Koombhongse S. (2000). Bending instability of electrically charged liquid jets of polymer solutions in electrospinning. J. Appl. Phys..

[12] Burnett L.R. (1949). Outlines for Guidance: The physician in the health program for secondary schools. Am. Phys. Educ. Rev..

[13] Zeng Y.C., Wu Y., Pei Z.G., Yu C.W. (2006). Numerical approach to electrospinning. Int. J. Nonlinear Sci. Numer. Simul..

[14] Wu S., Wang B., Ahmad Z., Huang J., Chang M.-W., Li J.S. (2017). Surface modified electrospun porous magnetic hollow fibers using secondary downstream collection solvent contouring. Mater. Lett..

[15] Lubasova D., Martinova L. (2011). Controlled morphology of porous polyvinyl butyral nanofibers. J. Nanomater..

[16] Bolelli K., Yalcin I., Ertan-Bolelli T., Özgen S., Kaynak-Onurdag F., Yildiz I., Aki E. (2012). Synthesis of novel 2-[4-(4-substitutedbenzamido/phenylacetamido)phenyl]benzothiazoles as antimicrobial agents. Med. Chem. Res..

[17] Kulandaivelu K., Mandal A.K. (2016). Positive regulation of biochemical parameters by tea polyphenol encapsulated solid lipid nanoparticles at in vitro and in vivo conditions. IET Nanobiotechnol..

[18] Liang J., Yan H., Puligundla P., Gao X., Zhou Y., Wan X. (2017). Applications of chitosan nanoparticles to enhance absorption and bioavailability of tea polyphenols: A review. Food Hydrocoll..

[19] Khakestani M., Jafari S.H., Zahedi P., Bagheri R., Hajiaghaee R. (2017). Physical, morphological, and biological studies on PLA/nHA composite nanofibrous webs containing Equisetum arvense herbal extract for bone tissue engineering. J. Appl. Polym. Sci..

[20] Rezaei B., Ghani M., Askari M., Shoushtari A.M., Malek R.M.A. (2016). Fabrication of thermal intelligent core/shell nanofibers by the solution coaxial electrospinning process. Adv. Polym. Technol..

[21] Jin Y., Nie J., Zhou Y., Yang D. (2008). Preparation of core-shell structured CS/PVA-PPC fibers by coaxial electrospinning. Acta Polym. Sin..

[22] Kumar G.G., Lee D.N., Kim P., Nahm K.S., Nimmaelizabeth R. (2009). Poly(vinylidene fluoride-co-hexa fluoropropylene)/poly vinyl alcohol porous membranes for the application of fuel cells. J. Polym. Res..

[23] Xu L., Si N., Liu H.Y., Tang X.P. (2014). Effect of flow rate on diameter of electrospun nanoporous fibers. Therm. Sci..

[24] Jiang H., Hu Y., Zhao P., Li Y., Zhu K. (2006). Modulation of protein release from biodegradable core–shell structured fibers prepared by coaxial electrospinning. J. Biomed. Mater. Res. Part B.

[25] Wang C.Y., Liu J.J., Fan C.Y., Mo X.M., Ruan H.J., Li F.F. (2012). The effect of aligned core–shell nanofibres delivering NGF on the promotion of sciatic nerve regeneration. J. Biomater. Sci. Polym. Ed..

[26] Dror Y., Salalha W., Avrahami R., Zussman E., Yarin A.L., Dersch R., Greiner A., Wendorff J.H. (2007). One-step production of polymeric microtubes by co-electrospinning. Small.

[27] Xu L., Si N., Lee E.W.M. (2013). Effect of humidity on the surface morphology of a charged jet. Heat Transf. Res..

[28] Pratyush D., Liu J., Satish K.A., Kyu T. (2007). Experimental and theoretical investigations of porous structure formation in electrospun fibers. Macromolecules.

[29] Liang J., Li F., Fang Y., Yang W., An X., Zhao L., Xin Z., Cao L., Hu Q. (2011). Synthesis, characterization and cytotoxicity studies of chitosan-coated tea polyphenols nanoparticles. Colloids Surf. B Biointerfaces.

[30] Xu Z., Xiong H.M., Wei X., Chen J. (2003). 12-Tungstosilicic acid doped polyethylene oxide as a proton conducting polymer electrolyte. Mater. Chem. Phys..

[31] Xu J., Zhang J., Gao W., Liang H., Wang H., Li J. (2009). Preparation of chitosan/PLA blend micro/nanofibers by electrospinning. Mater. Lett..

[32] Yu X., Xiang H., Long Y., Zhao N., Zhagn X., Xu J. (2010). Preparation of porous polyacrylonitrile fibers by electrospinning a ternary system of PAN/DMF/H_2_O. Mater. Lett..

[33] Ji L., Saquing C., Khan S.A., Zhang X. (2008). Preparation and characterization of silica nanoparticulate-polyacrylonitrile composite and porous nanofibers. Nanotechnology.

[34] Hou Z., Li C., Ma P., LI G., Zhang Z., Peng C., Yang D., Yang P., Lin J. (2011). Electrospinning preparation and drug-delivery properties of an up-conversion luminescent porous NaYF4:Yb3+, Er3+ @silica fiber nanocomposite. Adv. Funct. Mater..

[35] Xu L., Si N., Liu H.Y. (2014). Fabrication and characterization of Chinese drug-loaded nanoporous materials. J. Nano Res..

[36] Sedghi R., Shaabani A. (2016). Electrospun biocompatible core/shell polymer-free core structure nanofibers with superior antimicrobial potency against multi drug resistance organisms. Polymer.

[37] Sofokleous P., Stride E., Edirisinghe M. (2013). Preparation, characterization, and release of amoxicillin from electrospun fibrous wound dressing patches. Pharm. Res..

